# Photoinduced Electron Transfer in Organized Assemblies—Case Studies

**DOI:** 10.3390/molecules27092713

**Published:** 2022-04-22

**Authors:** Antonio Santoro, Giovanni Bella, Ambra M. Cancelliere, Scolastica Serroni, Giuliana Lazzaro, Sebastiano Campagna

**Affiliations:** Department of Chemical, Biological, Pharmaceutical and Environmental Sciences, University of Messina, Via F. Stagno d’Alcontres 31, 98166 Messina, Italy; giovanni.bella@unime.it (G.B.); ambramaria.cancelliere@unime.it (A.M.C.); sserroni@unime.it (S.S.); giuliana.lazzaro@studenti.unime.it (G.L.)

**Keywords:** electron transfer, artificial photosynthesis, supramolecular assemblies, donor-bridge-acceptor system, transient absorption spectroscopy

## Abstract

In this review, photoinduced electron transfer processes in specifically designed assembled architectures have been discussed in the light of recent results reported from our laboratories. A convenient and useful way to study these systems is described to understand the rules that drive a light-induced charge-separated states and its subsequent decay to the ground state, also with the aim of offering a tutorial for young researchers. Assembled systems of covalent or supramolecular nature have been presented, and some functional multicomponent systems for the conversion of light energy into chemical energy have been discussed.

## 1. Introduction

If we were to sort chemical reactions by their importance for life, electron transfer (ET) would be the best candidate to be ranked first [1]. The reasons for its importance lie in the key role it carries out in major biological processes, such as the electron transport chain and photosynthesis, as well as artificial processes, such as information storage (i.e., photography) and energy conversion (batteries) [1]. It is worth noting that ET is ubiquitous in all the branches of chemistry, from organic and biochemistry [2,3,4,5] to physical and inorganic chemistry [6,7,8,9]. The systematic study of the ET processes can be fundamental both for basic knowledge and for strongly applicative reasons.

One of the goals of ET studies lies in defining guidelines to prepare long-lived charge-separated states, eventually slowing down charge recombination reactions. To this aim, various factors, such as the chemical nature of the donor and acceptor, their distance, the environment, the driving force of charge separation, and charge recombination, must be taken into consideration. From such assumptions, it is evident that to study ET processes, it is necessary to simplify the systems by minimizing their variables. The most used method to build a simplified system consists of the development and synthesis of species, in which the electron donor (D) and the electron acceptor (A) are covalently connected. The unimolecular nature of the so-developed species allows us to avoid the diffusional complications inherent to the analogous bimolecular ET processes of D and A [10,11,12,13,14]. The nature of the connection between such subunits can be either covalent or supramolecular and can be a direct connection or it can be spaced by a bridge (B) between the two molecular moieties. Another significant advantage of connected D-B-A systems is the possibility to study ET efficiency based on the distance between the two subunits, which can be synthetically controlled by varying the length of the spacer B connecting the two subunits [15,16,17]. Moreover, in most of the studied cases, the bridge does not act as an inert subunit between donor and acceptor systems, but it can play an active role by accelerating or decelerating the electron transfer (and back electron transfer as well) [10,18]. So, on equal length, bridges of different natures can differently influence charge transfer and charge recombination.

In D-A or D–B–A systems, ET can be triggered by applying some kind of stimulus, such as thermal, electrochemical, or through the widely employed light excitation (photoinduced electron transfer, PET). The understanding of the mechanism that drives the PET and the charge recombination represents the key to shedding light on the possible conversion of solar energy into chemical energy, that is, photosynthesis. Actually, among all the forms of renewable energy, solar energy is definitely the most viable one, as follows: in just over one hour, the energy coming from our star and hitting the earth’s surface would be enough to cover society’s yearly energy demands [19]. With this picture in mind, the most urgent issue becomes how to harvest and store solar energy in a convenient way. To face these questions, the following two different technologies can be foreseen: artificial photosynthesis and photovoltaics. Although the latter is right now technologically much more advanced and is the only option industrially available, it shows many issues still unsolved, among which the biggest limit is constituted by the nature of the stored energy. In fact, in such a context, electricity is probably not the ideal option, because it requires batteries, which are expensive, hard to recycle, and can only store a limited amount of energy compared with their weight. Once again, natural emulation can be the best option to follow. By mimicking the mechanism of natural photosynthesis, it is possible in principle to take advantage of sunlight to convert low-energy content materials to high-energy content materials (fuels). Many are the possibilities in this area, spanning from water splitting with the production of molecular hydrogen and oxygen to ammonia or carbon monoxide [20,21,22,23,24,25]. Among the above-mentioned options, water splitting presents several advantages, and molecular hydrogen can be considered one of the possible candidates to replace fossil fuels. The hydrogen molecule shows a high combustion enthalpy value [26,27,28,29]. The technologies to store and transport it are already available, and after the combustion reaction, it regenerates water, giving life to a virtuous and environmentally green cycle with no by-product generated. This justifies the large interest towards artificial photosynthesis.

**Figure 1:**
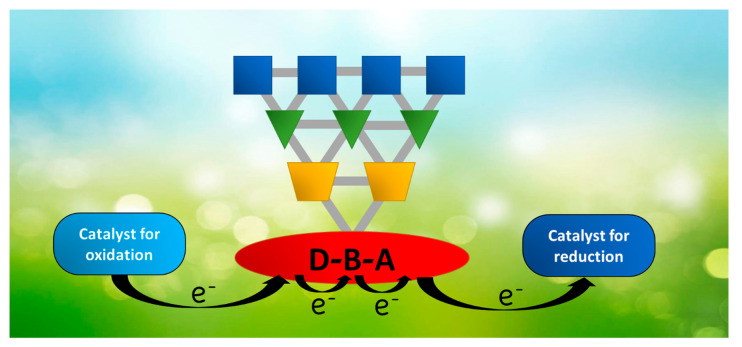
Schematization of an artificial photosynthetic system. The blue squares, green triangles, and orange pentagons constitute the antenna system; the red D-B-A subunit is the reaction center system.

An artificial photosynthetic system is basically constituted by a light-harvesting antenna system, a reaction center, and one or more catalysts for the oxidation/reduction of the involved species (H_2_O, CO_2_, etc.). The process starts when the sunlight is absorbed by the antenna, which normally consists of multiple chromophoric units to maximize the absorption spectrum, from UV to IR radiation. The antenna collects the electromagnetic energy, transforms it into electronic energy, and transfers it through a series of energy transfer steps to the reaction center. The latter species uses such energy to produce a photoinduced charge-separated state through one or more electron transfer processes by transforming electronic to redox energy. If the charge-separated state of the reaction center is sufficiently long-lived, it can transfer electrons or holes to the catalysts to start the multi-electron transfer processes and finally obtain a highly energetic species [16]. In Figure 1, the scheme of a water-splitting artificial photosynthetic system is described. The possibility to divide the whole mechanism of artificial photosynthesis into multiple single processes, so that they can be studied separately, allows us to reach encouraging results in this area. However, because of the complexity of the system, a fully functional device has not been presented yet. As a result of the reliance on diffusion-mediated processes, the non-assembled systems require photosensitizers with long-lived excited states, which brings us to the use of chromophores with heavy atoms, able to reach long-lived triplet states by intersystem crossing. At the same time, even the formed charge-separated state has to be long-lived since its interaction with catalysts is also diffusion dependent. Such a necessity brings along several disadvantages, such as the potential decomposition reaction of the systems, which is competitive with longer charge recombination or with the possible presence of singlet oxygen generated by the triplet state of the chromophore, and last but not least, the expensiveness of most of the heavy metals. In recent years, the covalent or supramolecular assembly of two or more components seems to be the way to obtain these kinds of devices more efficiently [30,31,32,33,34]. If on the one hand, such integrated systems show disadvantages, such as a more complex synthesis; on the other hand, the faster ET due to the direct connection between the components opens the gates to the use of a plethora of potential photosensitizers.

In this work, we will review some of the recent results obtained by our group in the field of photoinduced charge separation, particularly regarding new data showing the occurrence of photoinduced electron transfer in self-assembled species, which are relevant for solar energy conversion schemes. The review is also written to be useful as a tutorial for new researchers joining the field.

## 2. Fundaments of Electron Transfer in D-B-A Supramolecular Systems

### 2.1. Multicomponent Systems: Supramolecular vs. Molecular Entities

In his Nobel lecture of 1988 [35], Jean-Marie Lehn gave the definition of supramolecular chemistry, describing a supramolecular structure as the association of two or more chemical species held together by intermolecular forces. Starting from such a definition, multicomponent photophysical devices in which the components are held together by covalent interactions, such as the D-B-A systems, have a clear molecular nature. However, in the literature, there are plenty of papers describing this kind of structure [1] and cataloging them as supramolecular systems. The reasons for such an apparent misconception come from the existence of some features, proper supramolecular structures, which can be adopted by the D-B-A devices if designed in the correct way. In supramolecular architectures, the molecules are spatially organized to forge assembled systems able to show new features while maintaining the properties of the single components. Such peculiarities can also be found in multicomponent systems held together by covalent bonds, which can be defined as structurally molecular/covalent and functionally supramolecular [36]. The supramolecular feature discussed above constitutes a key aspect in the design of a working D-B-A device, in which it is mandatory to maintain the properties of the single components in the final assembled system in order to predict the behavior of the device in the design phase. Practically, the covalent connections have to guarantee a reduced electronic coupling between the subunits.

### 2.2. Photoinduced Charge Separation, Charge Recombination, and the Superexchange Mechanism

The choice of the correct building blocks and the ways to connect them is not only obviously essential to producing an operative D-B-A device, but it also determines the mechanism determining the photoinduced electron transfer happens. The photoinduced electron transfer can be oxidative, where the excitation of the D subunit leads to the transfer of an electron to the LUMO of the acceptor (Equations (1) and (2)), or reductive, where it is the excited state of the acceptor that provides the driving force for the electron transfer from the donor to the HOMO of the acceptor (Equations (3) and (4)).

(1) $$D-B-A\;+\mathrm{hv}\to D^{*}-B-A$$

(2) $$D^{*}-B-A\to D^{+}-B-A^{-}$$

(3) $$D-B-A\;+\mathrm{hv}\to D-B-A^{*}$$

(4) $$D-B-A^{*}\to D^{+}-B-A^{-}$$

The “supramolecular” identity of these systems allows one to thermodynamically estimate the driving force of both PET and charge recombination by exploiting the redox data of the single components. The Gibbs free energy in standard conditions and in an oxidative electron transfer pathway (similar treatment is possible to do for the reductive ET), assuming the validity of Koopman’s theorem [37], is expressed in Equation (5) as follows:

(5) ΔG0=e(E*ox−Ered)+W

where the term *E_ox_ is the oxidation potential of the excited state of the donor chromophore, *E_red_* is the first reduction potential of the acceptor, *e* is the charge of the electron, and *W* represents the work term, which is the difference between Coulombic stabilization energy of reactants and products, normally neglected in D-B-A systems [38,39]. In Equation (5), the term e*E_ox_ is described by the following equation:

(6) eE*ox=eEox−E0−0

In Equation (6), the term *E*^0−0^ is the excited state energy and can be estimated from photophysical data; in particular, it corresponds to the emission maximum of the donor recorded at 77 K [16,40,41,42]. Whereas the thermodynamics of the system depends on the electrochemical properties of both the donor and acceptor systems, the predictability of the PET reaction rate appears much more complicated.

The rate constant of an ET reaction can be expressed by using Equation (7) as follows:

(7) $$k_{el}=\frac{4\pi^{2}}{h}{\left|H_{ET}\right|}^{2}FCWD$$

The $H_{ET}$ term indicates the electronic coupling between reactants and products in the electron transfer reaction (which will be extensively discussed later). The *FCWD* is the Franck–Condon weighted density of states [43,44,45,46,47,48], a nuclear term that takes into account the overlap between the vibrational wavefunctions belonging to the products and reagents. Such a factor can be expressed in a single-mode approximation with a quantum mode of frequency $\upsilon_{i}$ [49] as follows:

(8) $$FCWD={\left(\frac{1}{4\pi \lambda_{0}k_{B}T}\right)}^{\frac{1}{2}}\underset{m}{\sum }S^{m}\frac{e^{-S}}{m!}exp\left[-\frac{{\left(\Delta G^{0}+\lambda_{0}+mh\upsilon_{i}\right)}^{2}}{4\lambda_{0}k_{B}T}\right]$$

where *S* is the Huang–Rhys factor $S=\frac{\lambda_{i}}{h\upsilon_{i}}$, $\lambda_{i}$ represents the inner-sphere reorganization energy and $\lambda_{0}$ represents the outer-sphere reorganization energy [48]. The *FCWD* term can be simplified at a high-temperature limit by using the following equation:

(9) $$FCWD={\left(\frac{1}{4\pi \lambda k_{B}T}\right)}^{\frac{1}{2}}exp\left[-\frac{{\left(\Delta G^{0}+\lambda \right)}^{2}}{4\lambda k_{B}T}\right]$$

where $\lambda =\left(\lambda_{0}+\lambda_{i}\right)$. Although the *FCWD* term is important for the predictability of the rate constant of an ET process, the electronic coupling term *H_ET_* is more influenced by the nature of the bridge. The electronic term can assume different expressions depending on which mechanism is followed to obtain the electron transfer. Both oxidative and reductive PET can happen through a superexchange or incoherent charge transport (hopping) mechanism, where the latter is less common in D-B-A systems. The superexchange model works by assuming that the HOMO and LUMO of the bridge are energetically far apart from those of the D and A systems. In these conditions, bridge orbitals are involved in the formation of virtual states that electronically connect D and A subunits, according to the McConnell superexchange model [50]. Since the virtual states involved in the PET mechanism are different considering oxidative or reductive pathways, they will be discussed separately.

**Figure 2:**
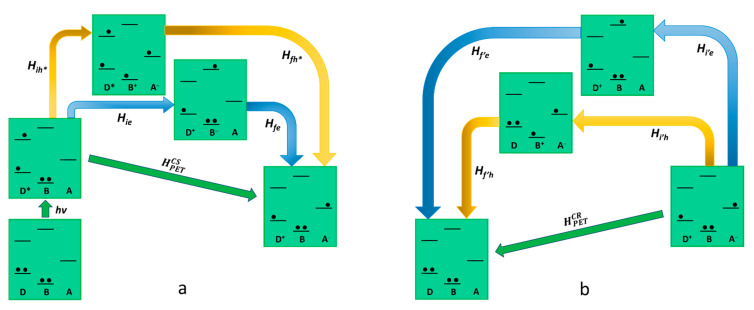
Schematic representation of oxidative photoinduced electron transfer in D-B-A dyads, according to superexchange. The states involved in the mechanism (virtual states pathways are described with blue and yellow arrows) are represented in terms of electronic configurations. On the left (**a**) (photoinduced) charge separation is described while charge recombination is shown on the right (**b**).

As far as the *Oxidative photoinduced electron transfer process* is concerned, the relevant electronic states involved (real and virtual) in the oxidative PET are represented in Figure 2. The initial state, where the donor is electronically excited, is coupled to the final charge-separated state through the following two possible virtual states: (i) the first concerns a virtual mono reduction of the bridge by the excited state of the donor, which assumes a positive charge (D^+^-B^−^-A); (ii) the second is made by the mono-electron-reduced acceptor, which take an electron from the bridge (D*-B^+^-A^−^). The superexchange electronic coupling matrix element for the oxidative PET process can be defined as follows:

(10) $$H_{PET}^{CS}=\frac{H_{ie}H_{fe}}{\Delta E_{e}}+\frac{H_{ih^{*}}H_{fh^{*}}}{\Delta E_{h^{*}}}$$

In Equation (10), all the Hamiltonian terms concern the electronic coupling between the real and the virtual state, as represented in Figure 2. The terms $\Delta E_{e}$ and $\Delta E_{h^{*}}$ represent the energy difference between the virtual state and the initial/final state for the electron and hole transfer, respectively (the energy of the initial and final state is equivalent as it refers to the transition state geometry). Considering Equation (10) for the case exemplified in Figure 2, the hole transfer mechanism (the second term of the sum), involving a higher energy virtual state, would be negligible. In light of this, in oxidative photoinduced electron transfer, the ET mechanism for the generation of the charge-separated state is usually the dominant one, and Equation (10) could be reported as $H_{PET}^{CS}=\frac{H_{ie}H_{fe}}{\Delta E_{e}}$. On the other hand, the charge recombination mechanism involves different virtual states compared to the ones involved in the forward photoinduced path since its final state does not coincide with the initial state of the charge separation. This means that in the electronic coupling matrix element for the charge recombination ($H_{PET}^{CR}$ in Equation (11)), both terms of the sum are valid to describe a possible pathway.

(11) $$H_{PET}^{CR}=\frac{H_{i^{\prime }e}H_{f^{\prime }e}}{\Delta E^{\prime }{}_{e}}+\frac{H_{i^{\prime }h}H_{f^{\prime }h}}{\Delta E^{\prime }{}_{h}}$$

Summing up, the only virtual state involved in the charge separation is the D^+^-B^−^-A, where the bridge is virtually mono-reduced, meanwhile, the possible virtual states in the charge recombination are D^+^-B^−^-A and D-B^+^-A^−^. We can conclude that in the design of a D-B-A system finalized to reach a long-lived charge-separated state via oxidative photoinduced electron transfer, it would be better to avoid the use of easy-to-oxidize bridge subunits. Such a kind of bridge would speed up the charge recombination without playing any effect on the charge separation.

**Figure 3:**
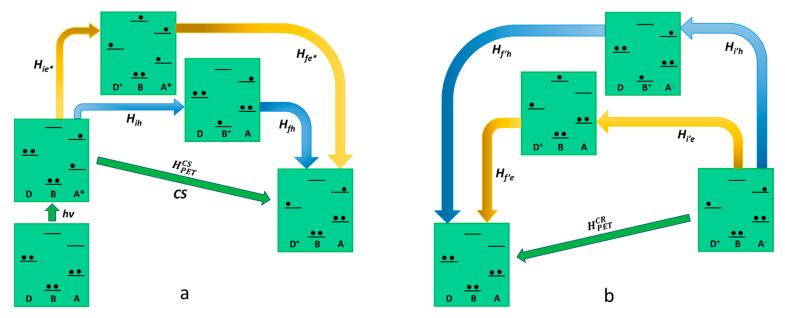
Schematic representation of reductive photoinduced electron transfer in D-B-A dyads, according to superexchange. The states involved in the mechanism (virtual states pathways are described with blue and yellow arrows) are represented in terms of electronic configurations. On the left (**a**) charge separation is described while charge recombination is shown on the right (**b**).

As far as *reductive photoinduced electron transfer* is concerned, also, in this case, there are two possible pathways that lead to charge separation with two virtual states involved, as described in Figure 3. One path contemplates the virtual state D-B^+^-A^−^, in which the excited state of the acceptor receives an electron from the bridge. The other virtual state is a D^+^-B^−^-A*, in which an electron is transferred from the ground state of the donor to the bridge. So, the superexchange electronic coupling matrix element for the reductive PET process can be expressed as follows:

(12) $$H_{PET}^{CS}=\frac{H_{ie^{*}}H_{fe^{*}}}{\Delta E_{e^{*}}}+\frac{H_{ih}H_{fh}}{\Delta E_{h}}$$

Different from the oxidative PET, in reductive PET, it is the first term of Equation (12) that can be neglected, so the dominant pathway is the one that includes the D-B^+^-A^−^ virtual state, allowing us to present the Equation (12) in a simplified version $H_{PET}^{CS}=\frac{H_{ih}H_{fh}}{\Delta E_{h}}$. The charge recombination mechanism in the reductive PET, connecting the D^+^-B-A^−^ charge-separated state and the D-B-A ground state, provides two different pathways involving as many virtual states, the electron transfer D^+^-B^−^-A and the hole transfer D-B^+^-A^−^. In this case, the electronic coupling matrix element for the charge recombination is formally equivalent to the one related to the oxidative PET as follows:

(13) $$H_{PET}^{CR}=\frac{H_{i^{\prime }e}H_{f^{\prime }e}}{\Delta E^{\prime }{}_{e}}+\frac{H_{i^{\prime }h}H_{f^{\prime }h}}{\Delta E^{\prime }{}_{h}}$$

As it results clearly, in the case of reductive PET, the conclusions are the opposite if compared to the oxidative mechanism. In this case, in fact, the only virtual state involved in the charge separation (D-B^+^-A^−^) shows an oxidized bridge, whereas both the possible virtual states (D^+^-B^−^-A and D-B^+^-A^−^) are accessible in the charge recombination step. We can conclude that in the design of this kind of device for a reductive PET, an easily reducible bridge should not be taken into consideration to avoid the acceleration of the charge recombination without producing the same effect in the charge separation step.

## 3. Examples of Photoinduced Electron Transfer Processes Recently Studied in the Messina Team

### 3.1. Covalently-Linked Multicomponent Species

From the general introduction discussed in the previous chapter, it appears clear that there are many variables that determine the possible kinetic mechanism and rate of the photoinduced charge separation and recombination. Some of the selected examples will be discussed in this section to illustrate the structural and energetic roles performed by the subunits and to exemplify the approach to the study of these systems.

**Figure 4:**
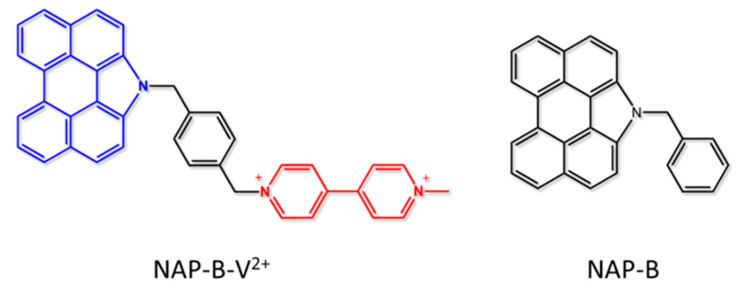
(**left**) D-B-A system constituted by a N-annulated perylene subunit as donor (NAP), an aromatic central moiety used as a bridge (B), and a methylviologen derivative as acceptor (V^2+^); (**right**) NAP-B model compound.

**Figure 5:**
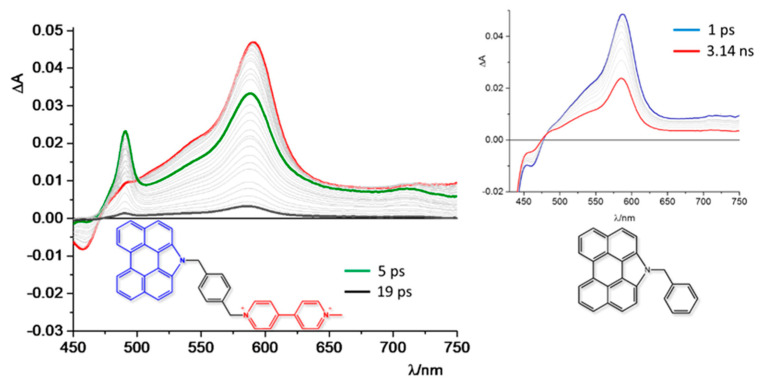
(**left**) Transient absorption spectra of NAP-B-V^2+^ dyad in acetonitrile; red spectrum was registered immediately after the pump, and time delays for the green and black spectra are given in the panel. (**right**) Transient absorption spectra of NAP-B registered at different delays, indicated in panel.

The first example presented consists of NAP-B-V^2+^, a fully organic D-B-A system made by a potentially strongly emissive N-annulated perylene subunit, connected through an aromatic bridge to a methylviologen electron acceptor derivative (Figure 4, left) [51]. The two methylene groups connected to the aromatic ring of the bridge were designed to interrupt the electronic communication between the D and A subunits, which allows threatening such a device as “supramolecular”. The “supramolecular” nature of this kind of system requires them to simplify their study by investigating the behavior of the single components separately. With this in mind, a model compound (NAP-B, Figure 4, right) was synthesized to study the spectroscopic and electrochemical behavior of the donor species. In fact, the first step consists of the comparison between the absorption spectra and redox potential of NAP-B-V^2+^ and NAP-B (Table 1) to verify that the connection between donor and acceptor subunits does not appreciably change the ground state properties (i.e., absorption spectra and redox data). In Table 1, it is possible to observe that the absorption and redox data of the two compounds are very close to each other, whereas the D-B-A system does not show luminescence at all, indicating that the strong emission of the “NAP-B” subunit in NAP-B-V^2+^ is fully quenched by the methylviologen acceptor. It is necessary to verify that the quenching of D-B-A luminescence is effectively due to the formation of a charge-separated state. Taking advantage of spectroscopic and redox data, the driving force of the photoinduced electron transfer, calculated by using Equations (5) and (6) and assuming that the work term is negligible, was $\Delta G_{PET}^{0}=-1.59\;\mathrm{eV}$, showing that the PET is largely allowed from a thermodynamic point of view. A further step consists of characterizing the transient species. For this reason, it is fruitful the use of pump-probe transient absorption spectroscopy combined with spectro-electrochemical analysis. In fact, for a correct interpretation of the transient spectra, it is essential to compare them with the absorption spectra of both the reduced viologen and the oxidized N-annulated perylene, to have a clear view of which species are forming during the decay and their timescale. Figure 5 shows the transient absorption spectra (TAS) registered in the same experimental conditions in acetonitrile of NP-B-V^2+^ and NP-B. The TAS registered just after the excitation is the same for both the compounds, with the transient absorption and bleaching bands attributed to the excited state of the perylene unit. After a few picoseconds, the trend changes, with the spectrum of the model NP-B compound showing a monotone decay to the ground state (interestingly, a sort of isosbestic is formed at Δ A=0, indicating that the initially-formed excited state directly decays to the ground state, without any intermediate state formation), whereas the NP-B-V^2+^ spectrum shows an increment of the absorption in the region around 490 nm, which was assigned to the formation of the radical cation of the N-annulated perylene after comparison with spectroelectrochemical data, with a time constant of 5 ps [51]. Unfortunately, in this case, it was not possible to observe the formation of the methylviologen radical anion because its absorption range was covered by the more intense absorption of the donor radical cation. The deactivation decay of the excited state was nevertheless assigned to the formation of the charge-separated state due to the clear evidence of the formation of the perylene cation and the strong driving force calculated for the photoinduced charge separation. The charge-separated state lifetime was calculated to be relatively short (19 ps), probably due to the driving force of the charge recombination ($\Delta G_{CR}^{0}=-1.29\;\mathrm{eV}$), combined with other factors such as reorganization energy, which are more difficult to calculate theoretically.

**Figure 6:**
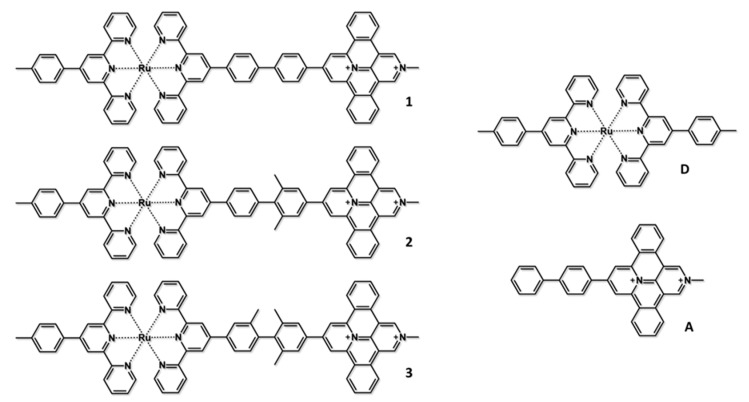
(**left**) Structural formulas of 1, 2, and 3 bichromophoric species systems; (**right**) the model compounds concerning the donor (D) and acceptor (A) moieties.

Another significant example consists of three D-B-A dyads, which differ from each other by the presence of methyl substituents on the aromatic rings of the biphenylene bridge (Figure 6) [17]. In these systems, an inorganic Ru(II) bis-terpy chromophore (terpy = 2,2′:6′,2″-terpyridine) is connected to an expanded, fused bipyridinium chromophore through differently substituted biphenylene bridges. The peculiarity of these systems consists of choosing D and A subunits to show different absorption spectral regions. In fact, the fully organic acceptor subunit absorbs in the 350–450 nm spectral region, where the inorganic donor absorbs only weakly, and conversely, the ruthenium-based subunit shows an intense absorbance in the 460–550 nm region due to a spin that allows the metal to ligand charge transfer (MLCT), where the fused bipyridinium subunit does not absorb. Following the same logic as in the example discussed above, different model compounds (D and A in Figure 6) were synthesized to compare the experimental results obtained for the more complex dyads with the simpler models. The redox data of all the compounds are shown in Table 2. The oxidation potential involves the oxidation of the ruthenium bis-terpy subunit, while the reduction was assigned to the one-electron reduction of the bipyridinium moiety. Similar to what was shown in the previous example, the redox data of the three dyads and the model compounds are very similar, indicating that the electronic coupling between donor and acceptor in all the dyads is negligible, and the subunits maintain their identity in the supramolecular structure. Such information is also confirmed by the comparison of the absorption spectra of the dyads and the models, where the spectra of the three dyads can be obtained by overlapping the absorption spectra of the model containing the donor and the one containing the acceptor moiety [17].

As far as the luminescence data are concerned, the model compounds show emission in the expected region of the spectrum as follows: a relatively weak emission at 648 nm, typical to the ^3^MLCT of Ru(II) bis-terpyridine complex for D, and emission at 610 nm for A, due to a CT state involving the biphenylene subunit as the donor and the positively charged fully extended bipyridinium (FEBP) fragment as the acceptor. Conversely, the dyads do not exhibit any luminescence at room temperature, indicating that the decay of the excited states follows radiationless pathways to the ground state. Starting from the absorption and redox data discussed, the thermodynamic feasibility of the oxidative photoinduced electron transfer has been investigated, obtaining a value the $\Delta G_{PET}^{0}$ equal to ca. 0.30 eV for all the species by using Equations (5) and (6).

To have an idea of the pathway followed by the excited state of the dyads, the pump-probe transient absorption spectroscopy has been essential. By exciting the ruthenium bis-terpyridine subunit at 570 nm, the transient spectra of the three D-B-A systems were similar to the one obtained for the **D** compound, in which the photo-populated ^3^MLCT state returns to the ground state without showing any other intermediates. Whereas the shape of the spectra does not show appreciable changes, the difference between the model and the dyads lies in the decay time constant, which is reduced from 1 ns for the model compound to 40, 173, and 360 ps for **1**, **2**, and **3**, respectively. The reasons for such a difference lie in the formation of the charge-separated state D^+^-B-A^−^. The lack of the charge-separated state fingerprint in the transient spectra of the dyads (which consists of an increase of the absorption at ca. 530 nm, the absorption region of the mono-reduced bipyridinium chromophore) indicates that the charge recombination is faster than oxidative photoinduced charge separation, inhibiting the accumulation and the detection of the charge-separated state. The other important difference evidenced by the transient spectra is the decay time shown by the dyads. The three species exhibit the same donor-acceptor distance, but the bridges bear 0, 2, and 3 methyl groups for the compounds **1**, **2**, and **3**, respectively, so the energy of the LUMOs of the bridges is different, affecting the superexchange electronic coupling matrix element driving the photoinduced electron transfer process, as described in Equation (10) [17]. The influence of the various substituents on the electron density of the bridge, a key factor determining the energy of the virtual states involved in the superexchange-mediated electron transfer, is linked to their inductive effects and geometrical constraints, as reported in detail in the original reference [17].

**Figure 7:**
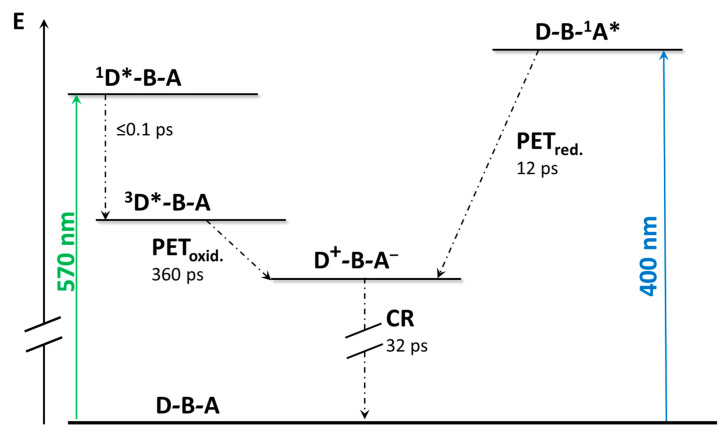
Schematization of the excited-state decay of **3**.

The panorama becomes more complex when the dyads are excited at 400 nm, which allows an almost selective excitation of the bipyridinium cation. In such a situation, the thermodynamic feasibility of a reductive photoinduced electron transfer increases by reaching a calculated value of $\Delta G_{PET}^{0}$ of ca. −1.06 eV for dyad **1** and ca. −1.18 eV for the other two species. The transient absorption spectra appear identical for the three dyads, evidencing the formation of the charge-separated state from the excited state of the acceptor subunit (D-B-A^*^ → D^+^-B-A^−^) after 15 to 32 ps, depending on the dyad considered. The evidence of this deactivation pathway foresees the presence in the transient spectrum of the absorption band of the mono-reduced species of pyridinium cation. The deactivation to the ground state happens with the same time constant obtained in the oxidative photoinduced electron transfer experiment, which confirms the presence of the charge separation also in the previous experiment. To the success in reaching the charge separation state in the reductive photoinduced electron transfer plays a key role in both the thermodynamic feasibility and the nature of the bridge, which, in agreement with the discussion explicated in the previous chapter, can speed up the reductive electron transfer rather than the oxidative one [17]. Quite interestingly, compounds **1**–**3** represent an example of systems in which the excited state decay depends on the excitation wavelength. Figure 7 shows the excited states and decays occurring in **3**.

**Figure 8:**
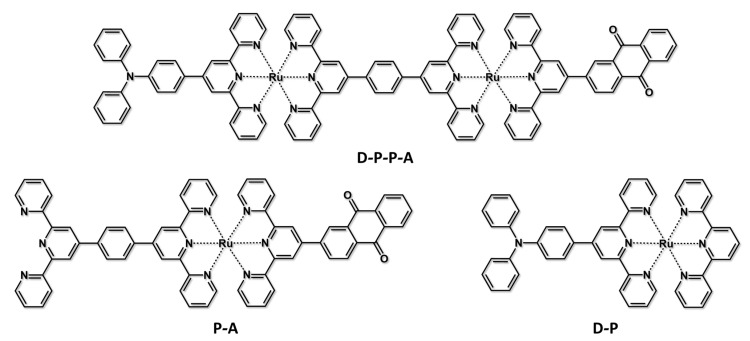
Structural formulae of D-P-P-A species and its model compounds P-A and D-P.

**Figure 9:**
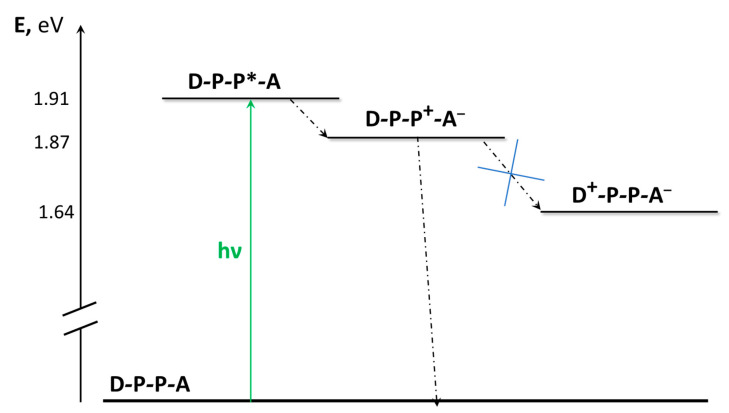
Energy levels and excited-state decays of D-P-P-A in mixed solvents. Dashed lines indicate proposed main radiationless decay routes.

A step further towards a consistent way to achieve a long-lived charge-separated state includes the development of triad (and in general, polyad) species, which in principle would allow further separation of the positive and negative charges, slowing down the charge recombination process. If at first sight, this looks like the “way to go”, it is undoubtedly a pathway hard to follow, particularly from a synthetic viewpoint. Many examples of these systems are present in literature [52,53,54,55,56,57,58,59,60]. Here we briefly present a triad constituted by two Ru(II)-based terpyridine-like chromophores interposed between a triphenylamine as an electron donor and an anthraquinone derivative as an electron acceptor, giving a linearly arranged D-P-P-A species [15], see Figure 8. Suitable model compounds are D-P and P-A. In an acetonitrile solution containing 1% of methanol, oxidative photoinduced electron transfer can be observed by transient absorption spectroscopy with a time constant of 380 ps, due to the formation of the D-P-P^+^-A^−^ species. However, although the formation of a “fully” charge-separated species (D^+^-P-P-A^−^) results in a thermodynamically accessible result of 0.23 eV, the kinetics of the charge recombination appears much faster, and no evidence of the charge-separated species was observed. A schematization of the excited-state levels and decays in D-P-P-A is shown in Figure 9.

**Figure 10:**
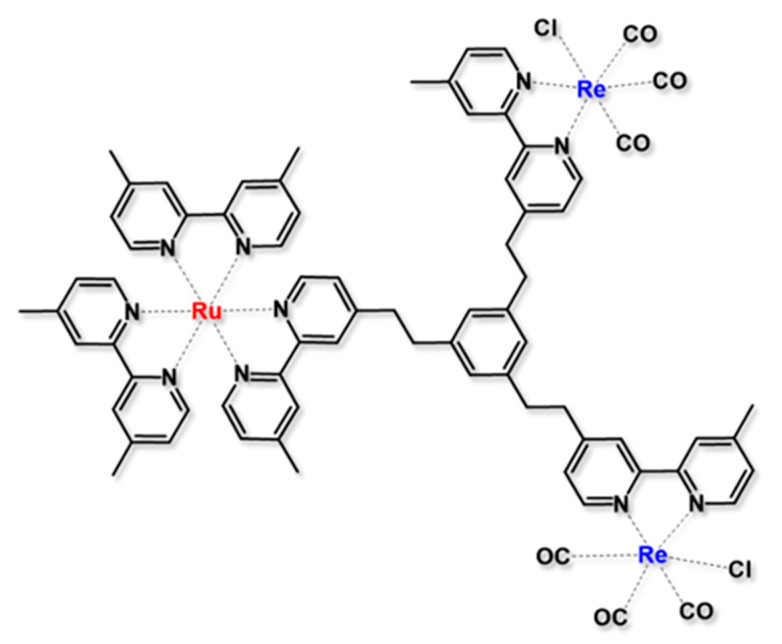
Structure of the supramolecular photocatalyst RuRe_2_ for CO_2_ reduction [61].

The concept of a donor-bridge-acceptor system, which was herein discussed with the aim of investigating the operational principles of photoinduced electron transfer, can be fruitfully used to design operative devices in many fields of application. In the following system, the D-B-A design was adopted to covalently connect through a proper bridge the photosensitizer and the catalyst in the design of a “supramolecular” photocatalyst for the CO_2_ to CO reduction, avoiding the kinetic limit of the diffusion speed [61,62,63]. For artificial photosynthesis purposes, a suitable photocatalyst that induces an efficient photocatalytic reaction via a reductive photoinduced electron transfer process should exhibit strong absorption in the region spectrum of solar light and should have a strong oxidation power in its excited state, and high stability in the one-electron reduced species. A Ru(II) based polypyridine complex can fulfill all the requirements needed, so it was used as a photosensitizer subunit, coupled through a tris-chelating polypyridine ligand (bridge unit) with two Re(I) diamine carbonyl complexes, acting as catalyst (RuRe_2_ in Figure 10) [61]. In RuRe_2_, the photosensitizer is energetically promoted to its excited state after the absorption of a photon and subsequently reduced by a sacrificial reductant in solution (a function that in regenerative photoelectrosynthetic cells should be performed by an electrode). The mono-reduced photosensitizer so generated can rapidly transfer an electron to the catalyst across the bridging ligand. Moreover, in this case, the bridge is crucial to maintaining the identity of the two connected species by interrupting the electronic connection. The bridge system used can achieve this task thanks to the presence of the methylene groups that interrupt the conjugation through the whole system; however, allowing the necessary electronic coupling to promote electron transfer. The multicomponent species RuRe_2_ showed a very efficient visible-light-induced catalytic CO formation with an outstanding value of turnover number (about 6000 in proper conditions), high selectivity for the CO production (over 95%), and high durability in the experimental conditions. Similar results are also reported for a similar trinuclear, in which the ratio between photosensitizer and catalyst units is changed from 1:2 as in RuRe_2_ to 2:1 [61].

### 3.2. Self-Assembled Systems

**Figure 11:**
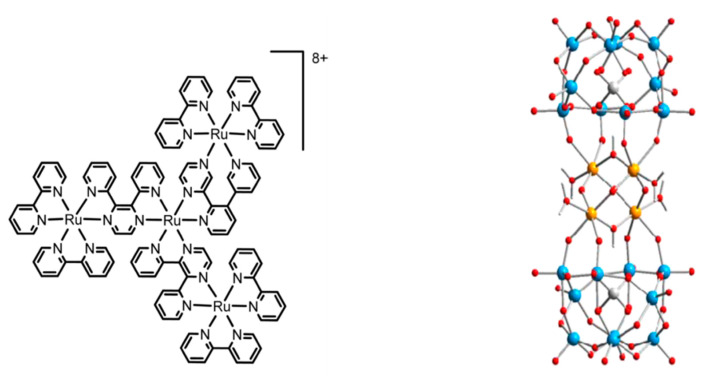
(**left**) Structural formula of [Ru{(μ-2,3-dpp)Ru(bpy)_2_}_3_]^8+^; (**right**) Structure of [Ru_4_(μ-O)_4_(μ-OH)_2_(H_2_O)_4_(γ-SiW_10_O_36_)_2_]^10−^.

As discussed in the introduction, the presence of a covalently linked bridge between donor and acceptor subunits overcomes the diffusion speed issue, paving the way for the adoption of a wide range of chromophores in the perspective of an efficient, long-lived charge-separated state. The same benefit provided by a covalently connected bridge can be obtained by using a supramolecular connection, which allows the formation of the device through the self-assembly process. Such systems, while presenting more difficulties to be studied, show some undeniable advantages, such as an easy way to be synthesized and the possibility to change the donor/acceptor ratio by simply changing their concentration in the solution. Although electron transfer in self-assembled systems is well known and studied [64,65,66,67], its use for operative artificial photosynthetic devices has a more recent origin [68]. The here presented self-assembled system [69], shown as an example, is composed of a tetranuclear dendrimeric Ru(II) polypyridine complex [Ru{(μ-2,3-dpp)Ru(bpy)_2_}_3_]^8+^ (where μ-2,3-dpp = 2,3-bis(2-pyridyl)pyrazine and bpy = 2,2′-bipyridine) [70] as the photosensitizer (Ru4) and the tetraruthenium polyoxometalate species [Ru_4_(μ-O)_4_(μ-OH)_2_(H_2_O)_4_(γ-SiW_10_O_36_)_2_]^10−^ (RuPOM) as the catalyst (Figure 11).

**Figure 12:**
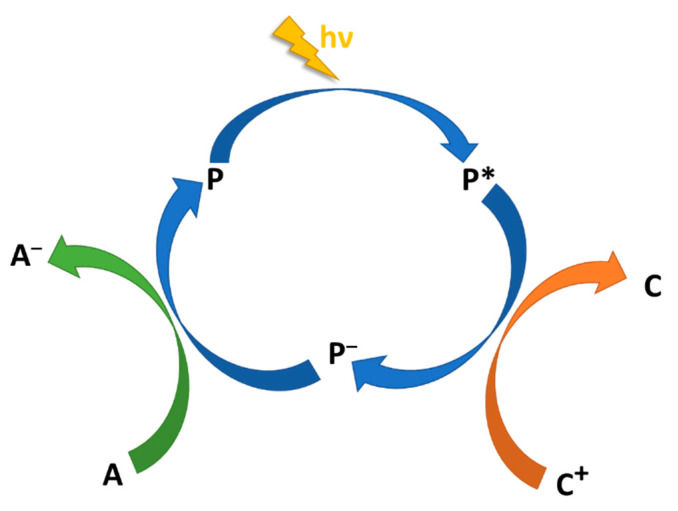
“Anti-biomimetic” scheme for catalyst oxidation performed by the Ru4/RuPOM system: reductive quenching of excited photosensitizer (P) by catalyst (C), followed by electron transfer to sacrificial acceptor (A) [69].

The target of such a system is the four-electron photooxidation of two water molecules, resulting in the formation of a new O=O bond and the release of four protons. Actually, water oxidation constitutes the real bottleneck toward artificial photosynthesis due to kinetic complications. Actually, photochemical water oxidation by the Ru4/RuPOM system, in the presence of persulfate as the sacrificial acceptor agent, takes place with an outstanding quantum yield of 0.30 and apparently is mainly limited by the sacrificial agent [70]. The sequence of electron transfer quenching events, as summarized in Figure 12, shows that the photoinduced excited state of the photosensitizer is first efficiently quenched by the catalyst through an intermolecular reductive electron transfer, generating an oxidized photocatalyst and a mono-electron-reduced photosensitizer [69]. The latter is subsequently regenerated by the sacrificial agent present in the solution (persulfate). Interestingly, the sequence of electron transfer processes leading to restoration of the photosensitizer and activation of the catalyst follows a route that is reversed with respect to the route used by natural photosynthetic organisms and by most of the artificial systems for photoinduced water oxidation as follows: In these latter systems, oxidative quenching of the photosensitizer usually occurs first (by sacrificial acceptors or intermediate acceptors), followed by hole transfer from the oxidized photosensitizer to the catalyst units. In the Ru4/RuPOM (plus sacrificial acceptor) system, an “anti-biomimetic” scheme is followed.

The driving force for self-assembly was mainly of electrostatic nature since the photosensitizer was 8+ and the catalyst was 10-. A conductimetric titration study was necessary to estimate the association equilibrium in the photocatalytic conditions (10 mM phosphate buffer pH 7), indicating the formation of ion-paired species with an average catalyst/photosensitizer ratio of 0.7, which is roughly in agreement with the charges of the components. In order to shed light on the mechanism and the kinetics, ultrafast transient absorption spectroscopy studies were performed [69]. The primary photochemical process, consisting of a reductive photoinduced electron transfer from the catalyst to the triplet excited state of the photosensitized, takes place rapidly in a few hundred picoseconds within ion-paired species (indeed, such a fast photoinduced electron transfer process could not be possible in a bimolecular fashion, limited by diffusion), followed by electron scavenging by the persulfate sacrificial acceptor, which competes with charge recombination (incidentally, this also indicates that persulfate is also most likely associated with the Ru4/RuPOM system). These findings clearly suggest a new way to combine photosensitizer and catalytic moieties, taking advantage of suitable chemical interactions and alternative photoinduced processes, for the development of efficient photoelectrosynthesis cells capable of overall water splitting. Recently, the anti-biomimetic pathway, detected for the first time in the Ru4/RuPOM system, has been successfully used in a photoelectrochemical cell for water oxidation [71].

## 4. Conclusions

Some examples of the photoinduced electron transfer processes occurring in selected multicomponent (supramolecular) species recently investigated by the Messina team have been reviewed, also with the aim of serving as a tutorial for young researchers joining the field. The reviewed systems range from a simple donor-acceptor species (the NAP-B-V^2+^ dyad) to triad donor-photosensitizer-acceptor systems (the D-P-P-A species, although in this case the fully-developed charge-separated species are not obtained), to multicomponent systems containing both metal-based and fully organic chromophores (the here called D-B-A systems, in which the organic chromophore also plays the role of electron acceptor). A multicomponent mixed-metal RuRe_2_ is also presented, in which photoinduced electron transfer drives the reduction of CO_2_ to CO with high turnover numbers and selectivity. Finally, a case of photoinduced electron transfer occurring in a self-assembled assembly made of a Ru(II)-based dendrimer as the photosensitizer and a ruthenium polyoxometalate species as the water oxidation catalyst is presented, highlighting the potential of electrostatic-driven aggregation for the preparation of functional systems for photochemical water oxidation.

## Figures and Tables

**Table 1:** Spectroscopical and electrochemical data of both the dyad NAP-B-V^2+^ and the model compound NAP-B.

	Absorption	Luminescence			Redox Data (V vs. SCE)	
	λ [nm] (ε[M^−1^cm^−1^])	λ [nm]	τ [ns]	φ	E_1/2(red)_	E_1/2(ox)_
**NAP-B-V^2+^**	422 (35,450)	-	-	-	−0.38	+0.89
**NAP-B**	421 (36,000)	435	5.0	0.9	-	0.88

**Table 2:** Redox data of the studied compounds.

	D	A	1	2	3
E_1/2(red)_ (V vs. SCE)	−1.27	−0.42	−0.42	−0.43	−0.45
E_1/2(ox)_ (V vs. SCE)	+1.22	-	+1.23	+1.23	+1.23
